# Newborn Nutritional Status at Birth and Its Association With Maternal Dietary Practices During Pregnancy in Gamo Zone, Southern Ethiopia: A Path Analysis

**DOI:** 10.1002/fsn3.71225

**Published:** 2025-11-14

**Authors:** Teshale Fikadu, Dessalegn Tamiru, Beyene Wondafrash Ademe

**Affiliations:** ^1^ School of Public Health, College of Medicine and Health Science Arba Minch University Arba Minch Ethiopia; ^2^ Department of Nutrition and Dietetics, Institute of Health Jimma University Jimma Ethiopia

**Keywords:** associated factor, dietary practice, pregnant women, stunting, wasting

## Abstract

Adequate dietary practice during pregnancy is central to improving pregnancy outcomes, maintaining maternal health, and supporting fetal growth and development. This study aims to determine the association between maternal dietary practices during pregnancy and newborn malnutrition at birth. A community‐based prospective cohort study was employed, enrolling 247 pregnant women with adequate and 248 with inadequate dietary practices. The test of proportion was used to determine the difference in stunting and wasting at birth among newborns of mothers with adequate and inadequate dietary practices during pregnancy. Path analysis was used to determine the causal direction, as well as the direct and indirect effects of covariates on length‐for‐gestational‐age and weight‐for‐length scores at birth. Betas (*β*) represent the coefficient that indicates the magnitude and direction of the relationship between the predictor and the outcome. And Confidence Intervals (CIs) are the values within which the true population coefficient is likely to lie. A range that includes zero indicates uncertainty. The overall proportion of stunting, wasting, and concurrent stunting and wasting at birth was 25.30% (95% CI: 21.66, 29.34), 10.32% (95% CI: 7.93, 13.34), and 1.82% (95% CI: 0.95, 3.47), respectively. Newborns from mothers with inadequate dietary practices had a significantly higher incidence of stunting (34.27%) and wasting (14.17%) at birth. Inadequate dietary practice (*β* = 0.48), better nutritional status (*β* = 0.10), decision‐making autonomy (*β* = 0.61), having dietary information (*β* =1.12), meal frequency (*β* = 0.11), socio‐economic status (*β* = 0.32), short stature (*β* = 0.01), and nutrient‐dense food intake (*β* = 0.10) and local leafy foods intake (*β* = 0.07), had significant effects on length‐for‐gestational‐age score. Inadequate dietary practice (*β* = 0.71), higher socio‐economic status (*β* = 0.08) and having dietary information (*β* = 0.67) had significant effects on weight‐for‐length score. Thus, governmental and nongovernmental organizations working on maternal and child health should address these factors to reduce the incidence and its short‐term and long‐term impacts of under nutrition at birth.

## Graphical abstract

Under nutrition, which includes conditions like stunting, wasting, and concurrent stunting‐wasting can start in the womb and is often prevalent at birth. Empowering women with autonomy in household decision‐making is essential for reducing the rates of under nutrition at birth, particularly in regions like Ethiopia, where mothers have limited influence over household and health service decisions. Adequate dietary practice during pregnancy is essential for enhancing the nutritional status of newborns and, consequently, reducing the intergenerational cycle of under nutrition.

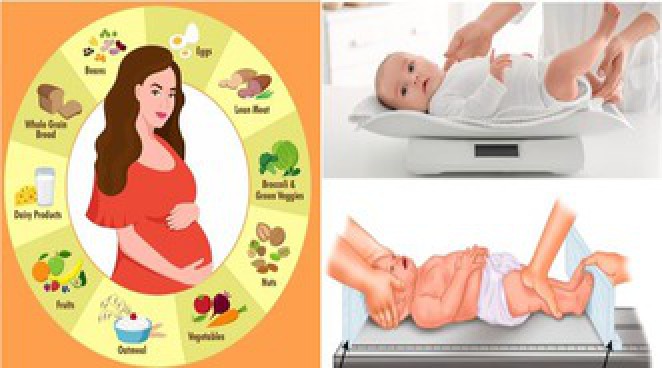

## 1 Introduction

Globally, a significant proportion of reproductive‐age women suffer from under nutrition, and a significant portion of the disease burden in children under the age of five is due to maternal and child under nutrition, mainly caused by inadequate dietary practices (Chea et al. 2023; UNICEF 2023). In low‐ and middle‐income countries, maternal under nutrition results in over 3.5 million deaths among mothers and children under the age of 5 years, as well as lasting physical and mental disabilities due to inadequate dietary intake during early life. Furthermore, it accounts for 800,000 neonatal deaths each year. Stunting, wasting, and deficiencies in micronutrients are estimated to contribute to nearly 3.1 million child deaths annually (Bhutta et al. 2013).

Globally, 51 million children under the age of 2 are affected by stunting (UNICEF 2023). Approximately half of these cases occur during pregnancy and in the first 6 months of life, a crucial time when infants depend solely on their mothers for nutrition. This highlights the importance of maternal dietary practices in reducing the prevalence and consequences of early‐life under nutrition (UNICEF 2023).

In low‐income countries, stunting and wasting continue to pose major public health challenges, with 4.7% of children suffering from concurrent stunting and wasting, which is associated with a 4.8‐fold increase in mortality risk (Victora et al. 2021). In Africa, the magnitude of stunting, wasting, and concurrent stunting and wasting at birth is also alarmingly high, ranging from 10% to 30.5% for stunting, 10.9% to 30% for wasting, and 1.2% to 2.5% for concurrent conditions (Gonete, Alemu, et al. 2021; Gonete, Kassahun, et al. 2021; Mwangome et al. 2019). This significantly raises the likelihood of neonatal mortality by 3.7 to 7.4 times (Ejigu and Tafese 2023). Despite a decline in the global prevalence of low birth weight, evidence indicates that both stunting and wasting are still common at birth (Victora et al. 2021).

Adequate dietary practice during pregnancy improves pregnancy outcomes, maintains maternal health, and supports fetal growth and development (Mazurkiewicz and Bronkowska 2021; Woldeamanuel et al. 2019) However, under nutrition during early life is linked to adverse health and economic consequences in adulthood, including shorter stature, reduced educational achievement, and lower income levels (Victora et al. 2021).

The dietary practices of most pregnant women in low‐ and middle‐income countries, such as Ethiopia, do not meet the recommendations set by the WHO and FAO and primarily rely on grain‐based foods, which are insufficient for proper fetal growth and development (Bitew et al. 2021). Dietary knowledge is essential for the intake of healthy, adequate, and high‐quality meals. However, in low‐ and middle‐income countries like Ethiopia, a significant number of pregnant women lack this knowledge, leading to inadequate dietary practices (Girma Tilahun et al. 2021; Yalewdeg et al. 2020). Inadequate intake of essential nutrients during pregnancy can increase the risks of maternal morbidity, fetal growth restriction (FGR), premature birth, low birth weight (LBW), and reduced gestational weight gain (Diddana 2019; Fall and Kumaran 2019; Mazurkiewicz and Bronkowska 2021; Seid et al. 2023). It may also result in abnormal growth of the placenta, impaired nutrient transfer from mother to fetus, endocrine disorder, and interfere with normal metabolic processes (Kuche et al. 2015; Wu et al. 2012). All these increase the risk of maternal and child malnutrition.

Dietary practices during pregnancy have a significant effect on stress responsiveness, cognitive abilities, and the overall health, growth, and development of both the mother and the fetus (Fall and Kumaran 2019; Mazurkiewicz and Bronkowska 2021; Woldeamanuel et al. 2019). Insufficient consumption of protein, carbohydrates, dairy, fruits, and vegetables can lead to reduced birth weight (Mohamed et al. 2022; Yonezawa et al. 2020) and a higher incidence of low birth weight (Quansah and Boateng 2020; Yonezawa et al. 2020), as well as increased rates of small for gestational age (SGA) and premature births (Madzorera et al. 2020). Furthermore, other studies indicate that a 3% rise in total protein intake is associated with a 19.4 g increase in birth weight, while a 3% increase in the consumption of animal source foods is associated with a 20.6 g increase in birth weight (Yang et al. 2022). Additionally, this increase in animal source food intake is associated with a 21% lower risk of low birth weight (LBW), a 13% lower risk of SGA, and a 14% lower risk of FGR (Yang et al. 2022).

Poor dietary habits during pregnancy raise the risk of under nutrition for both the mother and the fetus (Fikadu et al. 2024b; Zewude et al. 2024). Addressing malnutrition in the first 1000 days is crucial for survival, resistance to infections, and overall growth and development throughout life. However, dietary practices in this period are inadequate. This is evident from the low intake of diversified foods (Madzorera et al. 2020), frequent meal skipping, low consumption of animal‐source foods, and inappropriate child‐feeding practices (Seid et al. 2023; Tyagi 2023; Uwase et al. 2024; Victora et al. 2021).

In Ethiopia, some studies have explored the effect of dietary diversity on pregnancy and birth outcomes, as well as dietary practice during pregnancy (Bayked et al. 2024; Wondemagegn et al. 2022; Zerfu et al. 2016). However, evidence regarding the link between dietary practice and under nutrition at birth remains limited. Understanding dietary practices during pregnancy and their association with newborn nutritional status is crucial for mitigating health, growth, and developmental consequences for both mothers and offspring throughout their life. This study aims to provide valuable insights into dietary practices during pregnancy and their effects on newborn nutritional status, thereby assisting in the development and enhancement of nutrition intervention programs.

Adequate dietary practice is defined as the consumption of at least four meals daily, consumption of at least five out of ten food groups over the past 24 h, being categorized in the upper tercile for consumption of animal‐source food over the previous 7 days, and consuming food above the mean score over the previous 7 days. Inadequate dietary practice is defined as having at least one of the following: consumption of less than four meals daily, consumption of less than five out of ten food groups over the past 24 h, being categorized in the lower or middle tercile for consumption of animal‐source food over the previous 7 days, or consuming food below the mean score over the previous 7 days (Demilew et al. 2020; Fite et al. 2022a, 2022b).

## 2 Materials and Methods

### 2.1 Study Setting, Period and Design

The study was conducted in the Arba Minch Health and Demographic Surveillance Site (AM‐HDSS) found in Arba Minch Zurea and Gacho Baba districts of Gamo Zone southern Ethiopia. The provincial capitals, Arba Minch Zurea and Gacho Baba, are located 437 km and 439 km south of Addis Ababa, respectively. As of 2023/2024, the two districts have a total population of 164,629 (82,499 males and 82,630 females), projected from the 2007 CSA census data. Among them, 74,257 residents live in AM‐HDSS, which encompasses nine representative kebeles (the smallest administrative unit in Ethiopia), six in Arba Minch Zurea and three in Gacho Baba. In the surveillance site, it was anticipated that 2598 women would be pregnant in 2023/2024 and would access healthcare from seven nearby health centers, 37 health posts, and private healthcare facilities. A community‐based prospective cohort study design was employed from October 1, 2023, to December 1, 2024. The dietary habits of the area mainly focus on starchy staples, with key crops like maize, barley, and tubers like potatoes, sweet potatoes, and taro being commonly cultivated. Furthermore, the area produces a surplus of fruits, vegetables, and moringa stenopetala (Fikadu et al. 2024b).

### 2.2 Source and Study Population

All pregnant women who are permanent residents of AM‐HDSS were the source population; the source population for the exposed group included all mother‐newborn pairs with inadequate dietary practices during pregnancy, while the unexposed group comprised those with adequate dietary practices during pregnancy. All selected mother‐newborn pairs were the study population. However, women who were critically ill and those with known chronic illnesses such as HIV and diabetes were excluded during recruitment. Additionally, women with twin pregnancy (*n* = 1); miscarriages (*n* = 4); stillbirths (*n* = 4); and participants who moved (*n* = 3) outside the study areas were excluded. Participants were recruited at home between 16 and 20 weeks of gestation.

### 2.3 Sample Size Determination

The sample size is calculated using a two‐population proportion formula of equal sample size for the two groups using Epi‐Info7 statistical software version 7.2.3.1. Assuming a 95% confidence interval, 80% power, 8.2% expected proportion of stunting at birth among term newborns (outcome among unexposed) and a 17% expected proportion of stunting at birth among preterm newborns (outcome among exposed) (Sari and Sartika 2021), and an exposed to unexposed ratio of 1:1. Based on the above assumptions, the calculated sample size was 446. After adding 15% for possible loss to follow‐up, the total sample size was 512 (256 exposed and 256 unexposed).

### 2.4 Sampling and Sampling Procedure

The sample was proportionally allocated to all kebeles of AM‐HDSS based on the expected number of pregnant women. The community health extension worker listed the number of pregnant women between 16 and 20 weeks of gestation by confirming pregnancy using a digital fetal heart rate monitor in each kebele. Computer‐generated simple random sampling was used to recruit study participants. The selected pregnant women were evaluated for their dietary practices. Initially, pregnant women with adequate dietary practices (unexposed) were recruited, as the rate of adequate dietary practices during pregnancy was less than 50% (Nana and Zema 2018), followed by the nearest neighborhood pregnant women with inadequate dietary practices (exposed) for follow‐up.

### 2.5 Data Collection Tools and Procedures

The dietary practices were assessed using a validated food frequency questionnaire (FFQ) that featured 46 food items tailored to the local context. Pregnancy outcomes were tracked throughout gestation, and newborn anthropometric measurements were taken within 1 h of delivery.

Trained and experienced data collectors and supervisors were involved in the data collection and supervision. In addition, midwives working at nearby health centers collected the anthropometric data (Endline) within 1 h of delivery, while health extension workers followed the pregnancy outcomes throughout the gestation. The procedure for collecting baseline data at participants' homes was outlined in a previously published paper (Fikadu et al. 2024a, 2024b).

The health extension workers follow the pregnant women throughout their pregnancy with a tracing toolkit, to provide routine antenatal care, to assess any adverse pregnancy outcomes and link them to midwives at nearby health centers for delivery. They also identified participants who relocated to other areas and experienced abortions and stillbirths.

Midwives gather information on gestational age at delivery, sex of the newborn, birth weight, and birth length at the health facility within 1 h of delivery. Weights of the newborns were measured with a standard newborn scale to the nearest 1 g. The scale was adjusted to the zero level before weighing each newborn (Derbo and Debelew 2024; Girma et al. 2019).

The length of the newborn was measured by positioning the unclothed neonate in a supine position on the measuring board, supported gently by two individuals. One person ensured the head remained in contact with the headboard, while the other extended the leg by placing a hand on the knee, straightening it, and adjusting the footboard to make contact with the plantar surface of the foot, measuring to the nearest 0.1 cm (Wood et al. 2013). Digital open‐source toolkit (Kobo Collect) and validated tools were used to collect all data.

### 2.6 Operational Definition

#### 2.6.1 Still Birth

A baby born with no signs of life, after 28 weeks of gestation and before the complete expulsion of conception (World Health Organization 2014).

#### 2.6.2 Birth Weight

The weight of the newborn weighed within an hour of birth using a well‐calibrated beam balance scale (Gladstone et al. 2021).

#### 2.6.3 Wasting

Newborns whose weight‐for‐length < −2 SD (Villar et al. 2014).

#### 2.6.4 Stunting

Newborns whose length‐for‐gestational age < −2SD (Villar et al. 2014).

#### 2.6.5 Concurrent WaSt

Newborns with both wasted and stunted (Mwangome et al. 2019).

#### 2.6.6 Food Varity Score

Food varity score is calculated by summing all food items pregnant women consumed over the previse 7 days; then the mean was computed. Those pregnant mothers with FVS greater than the means categorized as having a “high” food variety score whereas those with below the means categorized as having “low” (Demilew et al. 2020; Fite et al. 2022a, 2022b).

#### 2.6.7 The Food Consumption Score (FCS)

The food consumption score is a sensitive measure of food frequency and dietary diversity, evaluated using WFP guidelines based on the previous 7 days of survey data. Each food item receives a score from 0 to 7, depending on how many days it was consumed. These items are categorized into groups, and the frequencies of all items within each group are added together. The FCS is computed by multiplying the frequency of each food group by its respective weight, and then summing these values to create a composite score. This score is classified as follows: Poor food consumption score ranges from 0 to 28, Borderline food consumption score ranges from 28.5 to 42, and Acceptable food consumption score is greater than 42 (Wiesmann et al. 2009).

Others, Meal frequency, Minimum dietary diversity for women (MDDW), Animal source food (ASF) consumption, Maternal dietary knowledge, Household wealth status, and Maternal nutritional status were described in detail in the previous papers (Fikadu et al. 2024a, 2024b).

#### 2.6.8 Nutrient‐Dense Food Consumption

Pregnant women who ate tubers (Potato, Sweet Potato, Taro, and Beetroot), vegetables (Collard Greens, Onion, Garlic, and Green Pepper), fruits (Banana, Mango, Avocado, and Papaya), meat (Raw Meat, Organ Meat, Fish, and Chicken), and eggs (Fikadu et al. 2024b).

#### 2.6.9 Leafy Local Food Consumption

Pregnant women who consumed Moringa stenopetala leaves and a locally prepared coffee leaf tea beverage (Fikadu et al. 2024b).

#### 2.6.10 Adequate Dietary Practice

Pregnant women who had at least four meals daily, high MDDW, high FVS, and high ASF consumption (Demilew et al. 2020; Fite et al. 2022a, 2022b).

#### 2.6.11 Inadequate Dietary Practice

Pregnant women who had at least one of less than four meals daily, low MDDW, low FVS, and low ASF consumption (Demilew et al. 2020; Fite et al. 2022a, 2022b).

### 2.7 Exposure Assessment

The dietary practices of the participants were evaluated during 16 to 20 weeks of gestation through home visits, using a modified and validated food frequency questionnaire (FFQ) that included 46 food items tailored to the local context and categorized into 10 food groups: (1) Cereal, Roots, and Tubers; (2) Pulses and Legumes; (3) Nuts and Seeds; (4) Dark Green Leafy Vegetables; (5) Vitamin A‐rich Fruits and Vegetables; (6) Meat, Fish, and Poultry; (7) Dairy and Dairy Products; (8) Eggs; (9) Other Fruits; and (10) Other Vegetables and a meal frequency questionnaire was administered for the previous week.

The number of food groups consumed by pregnant women in the previous 24 h was counted to analyze the Minimum Dietary Diversity for Women (MDDW). The Food Variety Score (FVS) was determined by counting the individual food items consumed by the women during a week, after which the mean FVS was computed. Animal source food (ASF) consumption is estimated by summing all animal source foods consumed by pregnant women in the past 7 days and converted in to terciles. Meal frequency, MDDW, FVS and ASF consumption were used to determine the exposure status.

### 2.8 Outcome Variables

The study focused on two outcome variables: stunting and wasting at birth. Stunting was measured using length‐for‐gestational‐age indices by INTERGROWTH‐21st, while wasting was assessed using weight‐for‐length indices by WHO AnthroPlus version 3.2.2 statistical software. One participant was excluded from the wasting analysis due to a birth length of less than 45 cm. To estimate gestational age, the first day of the last menstrual period (LMP) and ultrasonography measurements were used, while birth weight and length were measured following standard procedures.

### 2.9 Data Process and Statistical Analysis

The data were checked online and approved each day to ensure completeness and consistency. Once data collection was completed, it was downloaded in Excel format and imported into STATA 16.0 version statistical software for analysis.

A total of 495 participants, representing 96.7% of those at the beginning of the follow‐up, were included in the final analysis. At baseline, a total of 638 pregnant women were assessed for dietary practices. Of these, 256 women were classified as having adequate dietary practices (unexposed), while the nearest neighboring 256 pregnant women with inadequate dietary practices (exposed) were recruited. During the follow‐up period, among the exposed group, 8 participants were excluded (3 due to abortion, 2 due to stillbirth, 1 due to relocation, and 2 due to incomplete data). In the unexposed group, 9 participants were also excluded (1 due to twin pregnancy, 1 due to abortion, 2 due to stillbirth, 2 due to relocation, and 3 due to incomplete data).

Principal component factor (PCF) analysis was conducted to evaluate household socioeconomic status. Variables or assets held by more than 95% or less than 5% of the participants were excluded from the analysis, as they do not effectively differentiate between wealthier and poorer households. The assumptions for PCF were assessed using the Kaiser‐Meyer‐Olkin measure and Bartlett's test of sphericity. Subsequently, variables with cross‐loadings or complex structures were removed. Finally, three components which explained a total variance of 67.7% were extracted and the first component that explained the largest variance was used to categorize wealth status.

Similarly, PCF analysis and reliability analysis were employed to determine the dimensions and internal consistency of the items used to construct a composite score for maternal dietary knowledge. After excluding questions that violated PCF assumptions or displayed cross‐loadings or complex structures, the final 15 items, which showed adequate internal consistency (Cronbach's alpha = 0.74), were used to assess maternal dietary knowledge.

The test of proportion was used to compare the incidence of under nutrition (stunting/wasting) at birth between newborns whose mothers had inadequate dietary practices during pregnancy and those whose mothers had adequate dietary practices.

A structural path analysis model was employed due to the complex, and interconnected causes of malnutrition. Path analysis is a component of structural equation modeling that specifically deals with observed variables and facilitates the simultaneous analysis of both direct and indirect effects among independent and dependent variables (Grace and Bollen 2005). Initially, a hypothetical causal pathway diagram was created based on variables with a *p* < 0.25 from the bivariate analysis. Additionally, variables that showed significant associations in the previous literature review were considered to develop the path diagram (Harvey et al. 2022).

**FIGURE 1:**
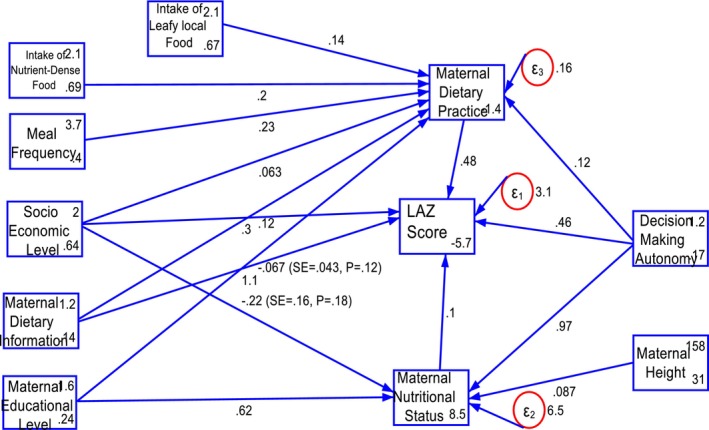
Structural path model with path coefficients predicting LAZ score at birth in AM‐HDSS; Gamo Zone, South Ethiopia.

**FIGURE 2:**
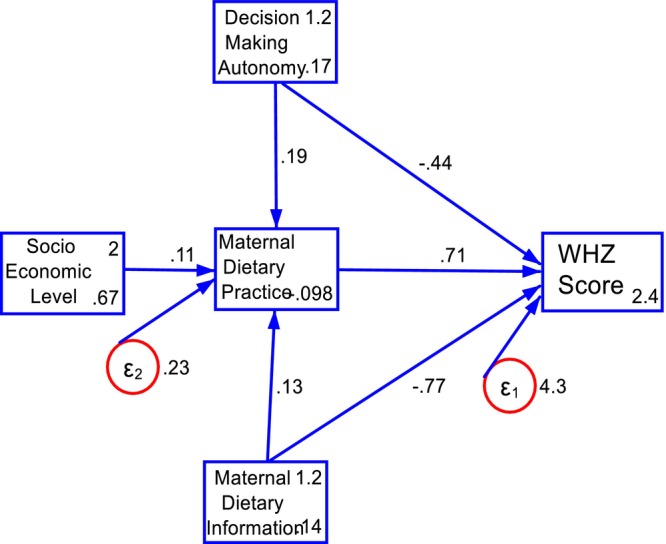
Structural path model with path coefficients predicting WHZ score at birth in AM‐HDSS; Gamo Zone, South Ethiopia.

Structural path analysis consists of a measurement model and a structural (or causal) model. The structural model represents a directional chain system, where single arrows indicate the causal relationships between variables (Mitku et al. 2023). The head of the arrow points to the effect, while the tail points to the cause, typically illustrated using path diagrams (Figures 1 and 2).

### 2.10 Assessment of Model Fit

Assumptions of multiple regression, such as normality, linearity, and multicollinearity, were checked, and the combined criteria for the goodness of fit for the structural path were applied (Dolatian et al. 2016; Singh et al. 2021), suggesting the best fit model (Table 1).

**TABLE 1:** Model fitness indices.

	*χ* ^2^/df	RMSEA	CFI	TLI	SRMR
Acceptable range	< 5	≤ 0.05	> 0.95	> 0.95	0.08
Length for gestational age	0.94	0.00	1.00	1.01	0.02
Weight for length	0.53	0.00	1.00	1.07	0.008

Abbreviations: CFI, comparative fit index; RMSEA, root mean squared error; SRMR , standardized root mean squared residual; TLI, Tucker‐Lewis index.

### 2.11 Ethical Consideration

The principle of the Declaration of Helsinki was used to conduct the study. Ethical approval and clearance were obtained from the Institutional Research Ethics Review Board (IRB) of Institute of Health, Jimma University under letter reference number (Nut/5029/2023). A cooperation letter was obtained from the Gamo Zone Health Office, and formal permissions were obtained from local administrators. Pregnant women were linked with healthcare facilities for antenatal care (ANC), where they received dietary counseling and nutritional education based on the WHO 2016 ANC guideline. Informed consent was obtained from the participants.

## 3 Results

There were no significant differences between women who had adequate versus inadequate dietary practices in aspects such as maternal age, place of residence, education level, family size, knowledge of nutrition, nutritional status, and height at the time of enrollment (Table 2).

**TABLE 2:** Baseline characteristics of study participants in AM‐HDSS; Gamo Zone; south Ethiopia.

Variable	Dietary practice		*p*
	Adequate, No (%)	Inadequate, No (%)	
Age of the respondent			
Less than 20 years	33 (13.36)	30 (12.10)	0.82
20 to 35 years	198 (80.16)	199 (80.24)	
36 years and more	16 (6.48)	19 (7.66)	
Place of residence			
Rural	207 (86.97)	230 (89.49)	0.26
Urban	31 (13.03)	27 (10.51)	
Religion			
Orthodox	45 (18.22)	55 (22.18)	0.27
Protestant	202 (81.78)	193 (77.82)	
Educational status			
Not attend formal education	77 (31.17)	98 (39.52)	0.06
Attend formal education	170 (68.83)	150 (60.48)	
Family size			
Less than five	110 (44.53)	123 (49.60)	0.26
Five and more	137 (55.47)	125 (50.40)	
Maternal nutritional knowledge			
Had poor knowledge	107 (43.32)	129 (52.02)	0.14
Had moderate knowledge	107 (43.32)	93 (37.50)	
Had good knowledge	33 (13.36)	26 (10.48)	
Number of pregnancy			
Primigravida	54 (21.86)	62 (25.00)	0.06
Multigravida	156 (63.16)	132 (53.23)	
Grand Multi	37 (14.98)	54 (21.77)	
Number of delivery			
Primipara	53 (28.49)	68 (34.00)	0.08
Multipara	118 (63.44)	106 (53.00)	
Grand multipara	15 (8.06)	26 (13.00)	
Had nausea vomiting			
Often	57 (23.08)	49 (19.76)	0.53
Some times	82 (33.20)	79 (31.85)	
No	108 (43.72)	120 (48.39)	
Alcohol consumption			
Yes	9 (3.64)	10 (4.03)	0.82
No	238 (96.36)	238 (95.97)	
Coffee consumption			
Yes	123 (49.80)	111 (44.76)	0.26
No	124 (50.20)	137 (55.24)	
Coffee leaf tea beverage consumption			
Yes	213 (86.23)	220 (88.71)	0.41
No	34 (13.77)	28 (11.29)	
Food aversion			
Yes	42 (17.00)	34 (13.71)	0.31
No	205 (83.00)	214 (86.29)	
Maternal MUAC in cm (mean ± SD)	24.08 ± 2.63	23.87 ± 2.51	0.37
Maternal height in cm (mean ± SD)	158.77 ± 5.41	157.99 ± 5.78	0.12

### 3.1 Magnitude of Newborn Under Nutrition at Birth and Its Link With Dietary Practices

The overall magnitude of stunting, wasting and concurrent stunting and wasting at birth was 25.30%, 10.32% and 1.82% respectively. The incidence of under nutrition (stunting/wasting) at birth was significantly higher among newborns whose mothers had inadequate dietary practices during early pregnancy compared to those whose mothers had adequate dietary practices (Table 3).

**TABLE 3:** Magnitude of newborn under nutrition at birth and its association with dietary practices during pregnancy in AM‐HDSS; Gamo Zone; south Ethiopia.

Dietary practice	Number of Participant	Incidence of stunting	95% CI	*p*
Length for gestational age *Z* score				
All participants	495	25.30%	21.66, 29.34	
Inadequate	248	34.27%	28.37, 40.18	0.001
Adequate	247	16.59%	11.96, 21.24	
Difference		17.68%	10.16, 25.19	
**Weight for length *Z* score**				
		**Incidence of wasting**		
All participants	494	10.32%	7.93, 13.34	
Inadequate	247	14.17%	9.82, 18.52	0.005
Adequate	247	6.48%	3.41, 9.55	
Difference		7.69%	2.37, 13.02	
**Concurrent stunting and wasting**				
All participants	494	1.82%	0.95, 3.47	

### 3.2 Direct, Indirect and Total Effects of Variables on LAZ‐Score and WHZ‐Score

Dietary practices, and nutritional status during early pregnancy had significant direct effects on the LAZ‐score, with coefficients of *β* = 0.48 (95% CI: 0.12, 0.84), and *β* = 0.10 (95% CI: 0.03, 0.17), respectively. Household socioeconomic status showed significant direct (*β* = 0.31; 95% CI: 0.08, 0.53) and total effects (*β* = 0.32; 95% CI: 0.09, 0.54) on the LAZ‐score. Maternal decision‐making autonomy demonstrated significant direct (*β* = 0.46; 95% CI: 0.02, 0.89), indirect (*β* = 0.16; 95% CI: 0.04, 0.27), and total effects (*β* = 0.61; 95% CI: 0.18, 1.05) on the LAZ‐score. Furthermore, dietary information during early pregnancy had significant direct (*β* = 1.07; 95% CI: 0.59, 1.54) and total effects (*β* = 1.12; 95% CI: 0.64, 1.60) on the LAZ‐score. Additionally, meal frequency (*β* = 0.11; 95% CI: 0.02, 0.20), the intake of nutrient‐dense foods (*β* = 0.10; 95% CI: 0.02, 0.18), the consumption of leafy local foods (*β* = 0.07; 95% CI: 0.01, 0.12), and maternal height (*β* = 0.01; 95% CI: 0.01, 0.02) had significant indirect effects on the LAZ‐score.

Dietary practices in early pregnancy have a significant direct impact on the WHZ‐score (*β* = 0.71; 95% CI: 0.34, 1.08). Furthermore, dietary information during this period demonstrated a significant direct (*β* = 0.77; 95% CI: 0.23, 1.25) and total effect (*β* = 0.67; 95% CI: 0.18, 1.16) on the WHZ‐score. Additionally, household socioeconomic status had a significant indirect effect (*β* = 0.08; 95% CI: 0.02, 0.13) on the WHZ‐score (Table 4).

**TABLE 4:** Direct, indirect and total effects of variables predicting newborn under nutrition at birth in AM‐HDSS; Gamo Zone; south Ethiopia.

Variables	Direct effect, *β* (95% CI)	Indirect effect, *β* (95% CI)	Total effect, *β* (95% CI)
Length for gestational age *z*‐score			
Dietary practice during pregnancy	0.48 (0.12, 0.84)**	NP	0.48 (0.12, 0.84)**
Maternal nutritional status	0.10 (0.03, 0.17)**	NP	0.10 (0.03, 0.17)**
Maternal decision making autonomy	0.46 (0.02, 0.89)*	0.16 (0.04, 0.27)**	0.61 (0.18, 1.05)**
Household socio economic status	0.31 (0.08, 0.53)**	0.01 (−0.04, 0.06)	0.32 (0.09, 0.54)**
Had dietary information	1.07 (0.59, 1.54)**	0.06 (−0.01, 0.1)	1.12 (0.64, 1.60)**
Meal frequency	NP	0.11 (0.02, 0.2)*	0.11 (0.02, 0.2)*
Maternal height	NP	0.01 (0.01, 0.02)*	0.01 (0.01, 0.02)*
Intake of leafy local food	NP	0.07 (0.01, 0.12)*	0.07 (0.01, 0.12)*
Intake of nutrient dense food	NP	0.10 (0.02, 0.18)*	0.10 (0.02, 0.18)*
Weight for length *z*‐score			
Dietary practice during pregnancy	0.71 (0.34, 1.08)**	NP	0.71 (0.34, 1.08)**
Had dietary information	0.77 (0.23, 1.25)**	−0.10 (−0.18, 0.001)	0.67 (0.18, 1.16)**
Household socio economic status	NP	0.08 (0.02, 0.13)**	0.08 (0.02, 0.13)**

Abbreviation: NP, no path.

* *p* < 0.05.

** *p* < 0.01.

## 4 Discussion

In this study, the incidence of stunting at birth was 25.30%. This finding is congruent with studies conducted in Northwest Ethiopia (Gonete, Alemu, et al. 2021; Gonete, Kassahun, et al. 2021) but is higher than those in Burkina Faso (Mwangome et al. 2019), Indonesia (Sari and Sartika 2021), and Bangladesh (Svefors et al. 2016). Conversely, it is lower than studies from South Ethiopia (Ejigu and Tafese 2023) and Pakistan (Harrison et al. 2020). The discrepancy is likely due to differences in study settings, socioeconomic and cultural factors, and variations in sample sizes.

The incidence of wasting at birth was 10.32%. This finding is comparable to a study conducted in Ethiopia (Gonete, Alemu, et al. 2021) and lower than a study from Burkina Faso (Mwangome et al. 2019). Likewise, the rate of concurrent stunting and wasting in the current study was 1.82%, which is consistent with findings from studies in northwest Ethiopia and Burkina Faso (Gonete, Alemu, et al. 2021; Mwangome et al. 2019). Potential reasons for these differences could include variations in dietary practices, study environments, and sample sizes.

The incidence of under nutrition at birth was closely linked to inadequate dietary practices during pregnancy and repeated maternal infections starting from conception (World Health Organization 2023). The current findings reinforce the idea that newborns whose mothers have inadequate dietary practices experience a significantly higher incidence of stunting and wasting at birth compared to those whose mothers have adequate practices (Table 2). This is a fact that the consumption of animal source foods (ASF), having a diverse diet (MDDW), and consuming more than three meals per day improve dietary intake in quality and quantities. This, enhances fetal growth and development (Kocyłowski et al. 2018; Mohamed et al. 2022) and reduces adverse pregnancy outcomes (Yang et al. 2023; Yonezawa et al. 2020), ultimately improving the nutritional status at birth.

The LAZ score and WHZ score of newborns from mothers with adequate dietary practices during early pregnancy were 0.48 and 0.71 units higher, respectively, than those from mothers with inadequate dietary practices. This is consistent with a study conducted in Iran, which indicated that a higher Dietary Diversity Score (DDS) during pregnancy was inversely correlated with nutritional status at birth (Karimi et al. 2022). Furthermore, research conducted in Tanzania revealed that a higher dietary intake was associated with a decreased risk of small‐for‐gestational‐age (Madzorera et al. 2020). Similarly, a study from Ethiopia found that inadequate dietary practices were associated with an increased incidence of under nutrition at birth (Ejigu and Tafese 2023). This is probably due to the bioavailability of nutrients in the maternal blood. Contrarily a randomized controlled trial in India showed that increased consumption of dairy products, fruits, and vegetables before and during pregnancy, using a specially designed snack, does not have an impact on birth weight (Potdar et al. 2014).

The fetus's nutrient intake relies on the mother's nutrient reserves and dietary habits. Early pregnancy is a critical period for placental and fetal growth, as this is when rapid placental development takes place. If a mother is malnourished during this phase, it can greatly affect fetal growth and result in under nutrition at birth (Bazer et al. 2004). This is supported by the current study indicating that the LAZ score of newborns increased by 0.1 for each centimeter rise in maternal MUAC during pregnancy. This finding is consistent with studies conducted in various regions of Ethiopia, which show that stunting at birth is more prevalent among undernourished mothers (Ejigu and Tafese 2023; Gonete, Kassahun, et al. 2021). Similarly, a study from Indonesia indicated that maternal nutritional status was directly related to birth weight (Yosefinata et al. 2022), while a study from Pakistan found no significant association (Sangi et al. 2021).

In this study, the LAZ score was 1.12 points and the WHZ score was 0.67 points higher among newborns of mothers who received dietary information during early pregnancy compared to those who did not. This is the fact that maternal dietary information, particularly during early pregnancy, is crucial for ensuring the consumption of healthy and adequate meals (Workneh et al. 2023). Improving dietary practices at this stage can contribute to a decrease in the occurrence of under nutrition at birth (Ejigu and Tafese 2023). Recent findings in Ethiopia show that there is a lack of dietary knowledge, information, and practices during pregnancy (Bayked et al. 2024; Girma et al. 2022), significantly impacting the nutritional status of newborns at birth.

Regular meals, improved appetite, satiety, intake of nutrient‐dense foods, diet quality (Fikadu et al. 2024a), and dairy consumption can reduce the incidence of malnutrition at birth. Similar findings were reported in the current study, which showed that the LAZ score of newborns increased by 0.11 for each additional meal consumed during early pregnancy. Additionally, the LAZ score was higher among newborns whose mothers consumed a higher tercile of nutrient‐dense foods and leafy local foods. This is likely due to the nutrient and antioxidant content of these foods (Fikadu et al. 2024a, 2024b). This is consistent with a study done in Ethiopia, in which the consumption of moringa leaves (local leafy foods in the study area) is associated with higher birth weights (Derbo and Debelew 2024). The antioxidants found in leafy local food support the digestive system, boost nutrient absorption, improve placental protein production, aid in the transfer of nutrients from mother to fetus, and lower oxidative stress (Derbo and Debelew 2024). Likewise, evidence indicates a nutrient‐dense diet during pregnancy lowers the rates of LBW, SGA, and FGR (Quansah and Boateng 2020; Yang et al. 2022), thereby decreasing the risk of malnutrition at birth.

Wealth status is a fuel to fulfill basic human needs and access to health services, balanced diet and consumption of high‐quality diet. The current study found that newborns from households with higher socioeconomic status had LAZ and WHZ scores that were 0.32 and 0.08 units higher, respectively, compared to those from households with lower socioeconomic status. This is a fact that households with higher socioeconomic status generally possess greater purchasing power, better access to food, and an increased chance of obtaining animal‐source foods. As a result, this contributes to improved maternal nutritional status, which ultimately reduces the incidence of malnutrition at birth. Similar evidence was reported in Japan, Indonesia, and China, where socioeconomic status was significantly associated with low birth weight (Lin et al. 2022; Okui and Nakashima 2022; Wulandari et al. 2022). However, evidence from Ethiopia indicated that socioeconomic status was not associated with malnutrition at birth (Gonete, Alemu, et al. 2021; Gonete, Kassahun, et al. 2021). This difference is likely due to variations in study settings; the former studies were facility‐based, while the current one is community‐based.

In this study, a 1 cm increase in maternal height corresponds to a 0.01 increase in the LAZ score. The finding is congruent with those studies conducted in different countries (Gonete, Kassahun, et al. 2021; Khatun et al. 2019; Solomons et al. 2015; Victora et al. 2015). This is probably due to short stature being an indicator of chronic under nutrition due to exposure to adverse environmental conditions from fetal life to adulthood. Mothers with chronic under nutrition often lack a well‐developed uterus and have a narrow pelvis, which can lead to fetal under nutrition and limited growth (Gonete, Kassahun, et al. 2021).

In this study, the LAZ score was 0.61 units higher among newborns of mothers who had decision‐making autonomy during early pregnancy compared to those who did not. This might be due to mothers who have decision‐making autonomy tending to use a continuum of maternal care, which increases their access to dietary information, healthy dietary practices, and maternal care during pregnancy (Shitie et al. 2020). As a result, this leads to better pregnancy outcomes, including enhanced nutritional status at birth. In contrast, women's autonomy in decision‐making is limited in low‐ and middle‐income countries, including Ethiopia, impacting health service utilization among women of reproductive age. This limitation puts them at risk of under nutrition, which, in turn, can lead to newborn malnutrition at birth (Fikadu et al. 2024b).

## 5 Strength and Limitation

The study's strengths included being conducted in a natural community setting and having a low loss to follow‐up. However, the lack of pre‐pregnancy nutritional status and anthropometric measurements may introduce intra‐ and inter‐observer bias, along with some limitations noted in earlier publications (Fikadu et al. 2024a, 2024b).

## 6 Conclusion

Dietary practice, as well as factors like maternal nutritional status, decision‐making autonomy, dietary counseling, meal frequency, household socio‐economic status, maternal short stature, and the intake of nutrient‐dense and locally sourced leafy foods, was found to be independent predictors of under nutrition at birth. Health authorities and concerned bodies should address these factors to reduce the incidence and both the short‐term and long‐term impacts of under nutrition in early life. Additionally, efforts should focus on empowering women in household asset generation and decision‐making. Enhancing access to dietary information is essential for improving dietary practices, nutritional health, and meal frequency.

## Author Contributions

**Teshale Fikadu:** conceptualization (equal), data curation (equal), formal analysis (equal), funding acquisition (equal), investigation (equal), methodology (equal), project administration (equal), resources (equal), software (equal), supervision (equal), validation (equal), visualization (equal), writing – original draft (equal), writing – review and editing (equal). **Dessalegn Tamiru:** conceptualization (equal), data curation (equal), formal analysis (equal), funding acquisition (equal), investigation (equal), methodology (equal), project administration (equal), resources (equal), software (equal), supervision (equal), validation (equal), visualization (equal), writing – original draft (equal), writing – review and editing (equal). **Beyene Wondafrash Ademe:** conceptualization (equal), data curation (equal), formal analysis (equal), funding acquisition (equal), investigation (equal), methodology (equal), project administration (equal), resources (equal), software (equal), supervision (equal), validation (equal), visualization (equal), writing – original draft (equal), writing – review and editing (equal).

## Conflicts of Interest

The authors declare no conflicts of interest.

## Data Availability

The data that support the findings of this study are available from the corresponding author upon reasonable request.
